# RANIBIZUMAB 0.5 MG TREATMENT IN ADOLESCENTS WITH CHOROIDAL NEOVASCULARIZATION: SUBGROUP ANALYSIS DATA FROM THE MINERVA STUDY

**DOI:** 10.1097/ICB.0000000000000825

**Published:** 2018-11-02

**Authors:** Philip G. Hykin, Giovanni Staurenghi, Peter Wiedemann, Sebastian Wolf, Shiao Hui Melissa Liew, Sabine Desset-Brethes, Harry Staines, Jun Li, Timothy Y. Y. Lai

**Affiliations:** * NIHR Biomedical Centre for Research in Ophthalmology, Moorfields Eye Hospital, London, United Kingdom;; † Department of Biomedical and Clinical Science Luigi Sacco, Luigi Sacco Hospital, University of Milan, Milan, Italy;; ‡ University of Leipzig, Klinik und Poliklinik für Augenheilkunde, Leipzig, Germany;; § Department Ophthalmology, Inselspital, Bern University Hospital, University of Bern, Bern, Switzerland;; ¶ Novartis Pharmaceutical Corporation, East Hanover, New Jersey;; ** Novartis Pharma AG, Basel, Switzerland;; †† Sigma Statistical Services, Balmullo, St Andrews, Scotland, United Kingdom; and; ‡‡ Department of Ophthalmology and Visual Sciences, The Chinese University of Hong Kong, Hong Kong Eye Hospital, Hong Kong, China.

**Keywords:** adolescents, anti–vascular endothelial growth factor therapy, best-corrected visual acuity, choroidal neovascularization, multicenter, open-label, ranibizumab

## Abstract

**Purpose:**

To evaluate the efficacy and safety of ranibizumab 0.5 mg in adolescent patients with any choroidal neovascularization etiology enrolled in the 12-month MINERVA study.

**Methods:**

In the open-label, non-randomized study arm, ranibizumab 0.5 mg was administered to five adolescents (aged 13–17 years). The findings were assessed descriptively as individual case reports at Month 12. Best-corrected visual acuity changes, central subfield thickness, treatment exposure, and safety were described over 12 months.

**Results:**

Baseline choroidal neovascularization etiologies of the study eye included choroidal neovascularization secondary to Best disease (n = 2), idiopathic chorioretinopathy (n = 2), and optic disk drusen (n = 1). At Months 2, 6, and 12, the observed mean best-corrected visual acuity changes in the study eye from baseline were +9.2, +16.6, and +16.6 letters, respectively, and the observed mean central subfield thickness change from baseline was −31.4, −87.6, and −116.4 *μ*m, respectively. Adolescent patients received a mean of three (range, 2–5) ranibizumab injections in the study eye. No adverse events or serious adverse events related to ranibizumab were reported.

**Conclusion:**

Ranibizumab 0.5 mg treatment was beneficial in improving visual acuity and stabilizing or reducing central subfield thickness in five adolescents with differing choroidal neovascularization etiologies requiring infrequent injection. No new safety findings were observed over 12 months.

## Abstract 2

In the 12-month MINERVA study, a subgroup of 5 adolescent patients aged 13–17 years received open-label ranibizumab 0.5 mg at baseline, followed by individualized pro re nata regimen based on disease activity, for the treatment of choroidal neovascularization. Visual and anatomical outcomes were improved, and no new safety findings were observed with ranibizumab.

Choroidal neovascularization (CNV) in children is a rare ocular disease that can cause significant visual impairment and even severe vision loss if untreated.^1^ In children, CNV may be associated with a specific underlying ocular condition, but in many cases, the cause remains uncertain.^1,2^ The overall prevalence of CNV is much lower in children and adolescents than in adults; however, the prevalence increases with age and remains a cause for significant decline in visual function.^3,4^

Currently, there is no approved therapy or established standard of care for the treatment of CNV in adolescents and children. The available treatment modalities for CNV in the pediatric population include observation, macular surgery, laser photocoagulation, verteporfin photodynamic therapy, and, more recently, the off-label use of anti–vascular endothelial growth factor (anti-VEGF) agents.^2,5,6^ Limited data on the natural history of CNV in adolescents suggest that observation alone may be required in some cases.^7^ Furthermore, although surgery may improve the visual acuity in such patients, there could be an inherent risk of ocular complications or the need for reoperation or postoperative laser treatment.^2,7^ Laser photocoagulation is reported to be safe but involves a risk of scarring and thermal injury.^2^ A small number of case reports and series have reported that verteporfin photodynamic therapy can improve visual acuity with immediate reduction in CNV leakage in children; however, retinal pigment epithelial atrophy has been reported.^2,3,8^

Ranibizumab is approved for the treatment of CNV secondary to age-related macular degeneration (AMD) and CNV in adults.^9^ The MINERVA study was specifically designed to evaluate the efficacy and safety of ranibizumab 0.5 mg using an individualized pro re nata regimen based on disease activity in adult patients with visual impairment due to CNV associated with any cause other than neovascular AMD and myopic CNV, with an open-label, nonrandomized setting in adolescent patients.^10,11^

Here, we present the efficacy and safety results of ranibizumab 0.5 mg in adolescent patients with CNV enrolled in the MINERVA study.

## Methods

### Study Design and Population

The MINERVA study was a 12-month, Phase III, randomized, double-masked, sham-controlled, multicenter study in adult patients, with a nonrandomized, open-label group of adolescent patients.^10,11^ Of the total 183 patients enrolled in this study, 178 were adults and 5 were adolescent.^10,11^ The study was conducted in accordance with the Declaration of Helsinki. Written informed consent from guardians of adolescent patients and written assent from adolescent patients were obtained before any study assessments were performed.

Treatment-naive adolescent patients aged ≥12 to <18 years with visual impairment due to any CNV etiology were included in the study. Adolescent female patients with positive pregnancy tests were excluded from the study. Complete eligibility criteria have been described previously.^10,11^

### Treatment

All adolescent patients received open-label ranibizumab 0.5 mg in the study eye at baseline, followed by an individualized pro re nata regimen from Month 1 onward based on the disease activity, as assessed by the investigator at each monthly visit. Evidence of disease activity was judged clinically (e.g., visual acuity impairment, intraretinal/subretinal fluid, and hemorrhage or leakage) or based on real-time imaging and functional testing. Retreatment was warranted by the presence of disease activity and as per the investigator's discretion. The fellow eye could receive ranibizumab treatment if it presented with or developed CNV due to the same underlying disease as in the study eye during the course of the study.

For adolescent patients, intravitreal injections were administered and anesthetic procedures were performed as per the local practice.

### Objectives

The objective of the study was to describe the efficacy and safety findings with ranibizumab 0.5 mg treatment in adolescents with any CNV etiology, similar to those assessed for adult patients over 12 months.^10,11^ The study assessments included the followings: 1) change in best-corrected visual acuity (BCVA) of the study eye from baseline to Months 2, 6, and 12; 2) change in central subfield thickness (CSFT) of the study eye from baseline to Months 2, 6, and 12; 3) change in macular volume of the study eye from baseline to Months 2, 6, and 12; 4) presence of subretinal fluid, intraretinal edema, and CNV leakage in the study eye at Months 2, 6, and 12; 5) treatment exposure in the study eye over 12 months; and 6) safety over 12 months.

### Efficacy and Safety Assessments

Study assessments were performed at screening, baseline (Day 1), and at all monthly visits up to the last visit. Spectral domain optical coherence tomography (OCT) including Cirrus (Carl Zeiss Meditec, Dublin, CA) and Spectralis (Heidelberg Engineering, Dossenheim, Germany) was performed at each monthly monitoring visit. The images were evaluated for quantitative (e.g., CSFT and macular volume) and qualitative (e.g., macular edema, cysts, and intraretinal and subretinal fluid) anatomical parameters and their change over time by the central reading center (CRC; Bern Photographic Reading Center, Bern, Switzerland) and by the investigators at the sites. The analysis is based on the assessment by the CRC. Macular volume was recorded by the CRC as the volume of 3-mm diameter field around the foveal center. Fluorescein angiography (FA) and color fundus photography assessments are described previously.^10,11^ Data were collected on the number of ranibizumab treatments received over 12 months.

Safety assessments included type, frequency, and severity of adverse events (AEs) and serious AEs, and the occurrence of abnormal vital signs or intraocular pressure ≥30 mmHg at any time point up to Month 12.

### Statistical Analysis

The efficacy and safety outcomes of the adolescent patients were assessed descriptively as individual case reports at Month 12. Descriptive statistics included the number of observations (n), mean, median, SD (as required), and ranges for continuous variables, and frequencies and percentages for categorical values. Statistical analysis was performed using SAS (version 9.3 or higher).

## Results

Five adolescent patients aged 13–17 years and diagnosed with CNV in the study eye were included in the study. At baseline, two patients had CNV secondary to idiopathic chorioretinopathy, two patients had CNV due to Best disease, and one patient had CNV secondary to optic disk drusen (Table 1). All patients completed the 12-month study.

**Table 1.** Patient Demographics and Baseline Ocular and Disease Characteristics

	Adolescent Patients				
	A	B	C	D	E
Demographics					
Age, years	17	15	14	13	14
Sex	Female	Female	Female	Male	Female
Race	White	White	White	White	White
Country	Germany	Germany	Germany	Poland	Turkey
Ocular and disease characteristics (study eye)					
BCVA, letters	34	82	63	65	46
CSFT, *μ*m	696	216	245	451	520
Time since diagnosis of current ocular condition/underlying disease, months	0/85	0/0	0/31	48/75	0/0
Baseline underlying disease	Best disease	Idiopathic	Drusen disk	Best disease	Idiopathic
Subretinal fluid	Definite	Definite	Absent	Definite	Definite
Intraretinal edema	Absent	Absent	Definite	Absent	Absent

### Efficacy

Best-corrected visual acuity improved from baseline to Month 12 in all 5 adolescent patients (Table 2). Baseline BCVA ranged from 34.0 to 82.0 letters (mean, 58.0 letters) and the change in BCVA from baseline to Month 12 ranged from +5.0 to +38.0 letters (mean, +16.6 letters). The mean change in BCVA of the study eye from baseline to Months 2, 6, and 12 was +9.2, +16.6, and +16.6 letters, respectively.

**Table 2.** Best-Corrected Visual Acuity and CSFT of the Study Eye

BCVA, Letters	Baseline	Month 2	Month 6	Month 12
Adolescent patients				
A	34	47	51*	55
B	82	84	91	87
C	63	73	77	77
D	65	71	73	70
E	46	61	81	84
Change in BCVA from baseline, letters				
Mean		+9.2	+16.6	+16.6
Min–max		+2 to +15	+8 to +35	+5 to +38

CSFT, *μ*m	Baseline	Month 2	Month 6	Month 12
Adolescent patients				
A	696	481	501*	490
B	216	220	222	226
C	245	246	247	254
D	451	529	453	342
E	520	495	267	234
Change in CSFT from baseline, *μ*m				
Mean		−31.4	−87.6	−116.4
Min–max		−215 to +78	−253 to +6	−286 to +10

Optical coherence tomography data for the study eye, as assessed by the CRC.

* Month-7 visit data reported here, as Month-6 visit was not reported for this patient.

One of the patients has received ranibizumab in the fellow eye (Patient A) for the treatment of CNV secondary to Best disease (the same CNV etiology as in the study eye). The fellow eye had BCVA gain of +12.0 letters at Month 12, from a baseline BCVA of 42.0 letters.

Over 12 months, CSFT was stable or reduced in all 5 adolescent patients. The mean change in CSFT of the study eye from baseline to Months 2, 6, and 12 was −31.4, −87.6, and −116.4 *μ*m, respectively (Table 2). In the majority of adolescent patients by Month 12, macular volume was reduced or stable, whereas subretinal fluid, intraretinal edema, and CNV leakage were absent. The OCT and FA findings (macular volume, subretinal fluid, intraretinal edema, and CNV leakage) of the study eye at baseline, Months 2, 6, and 12 are described in Table 3.

**Table 3.** Macular Volume, Subretinal Fluid, Intraretinal Edema, and CNV Leakage in the Study Eye

Adolescent Patients	Baseline	Month 2	Month 6	Month 12
Macular volume, μL				
A	3.73	2.82	2.70*	2.54
B	2.20	2.17	2.18	2.20
C	2.43	2.34	2.39	2.41
D	2.61	2.79	2.71	2.44
E	3.67	3.03	2.15	2.10
Subretinal fluid†				
A	Definite	Absent	Definite*	Definite
B	Definite	Absent	Absent	Absent
C	Absent	Absent	Absent	Absent
D	Definite	Definite	Definite	Definite
E	Definite	Definite	Definite	Absent
Intraretinal edema†				
A	Absent	Absent	Absent*	Absent
B	Absent	Absent	Absent	Absent
C	Definite	Absent	Definite	Definite
D	Absent	Absent	Absent	Absent
E	Absent	Absent	Absent	Absent
CNV leakage‡				
A	Definite	Absent	—	Absent
B	Definite	Absent	—	Absent
C	Definite	Definite	Definite	Definite
D	Definite	Absent	Absent	Absent
E	Definite	Definite	Absent	Absent

Macular volume was recorded by the CRC as the volume of 3-mm diameter field around the foveal center.

* Month-7 visit data reported here, as Month-6 visit was not reported for this patient.

† Optical coherence tomography, as assessed by the CRC.

‡ Fluorescein angiography, as assessed by the CRC.

**Fig. 1.**
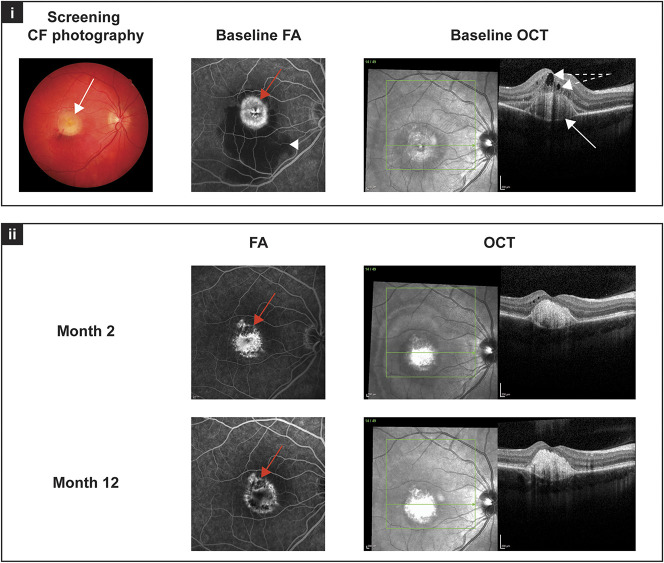
Image studies from “Patient A” at screening/baseline, and Months 2 and 12 (Table 1): (i) at baseline, the typical yellowish, egg-yolk–like, round and isolated lesion in the center of the macula is visible in the color fundus photograph (white arrow). The FA at baseline shows an active CNV with leakage (red arrow) as well as subretinal hemorrhage in the inferior part of the macula (white arrowhead). Optical coherence tomography scans at baseline reveal intraretinal and subretinal fluid (white arrows with broken line) as well as subretinal vitelliform material (white arrow). (ii) Fluorescein angiography images at Months 2 and 12 show a quiescent lesion with only staining (red arrow), and the subretinal hemorrhage has resolved. Optical coherence tomography scans at Month 2 show no subretinal fluid, the intraretinal fluid is improved, the foveal pit is visible, and subretinal hyperreflective material is present. At Month 12, the foveal structure is unchanged, there are some intraretinal cystoid changes, but no signs of CNV activity are visible on OCT scans.

As an example, the FA, OCT, and color fundus photography outcomes for one of the patients with CNV secondary to Best disease are shown in Figure 1.

### Treatment Exposure

Over 12 months, a mean of three ranibizumab injections (range, 2–5) were administered in the study eye out of possible 12 injections (Table 4). The patient who received treatment in the fellow eye for CNV secondary to Best disease (Patient A) received four ranibizumab injections in this eye at Months 3, 4, 7, and 10.

**Table 4.** Number of Injections in the Study Eye Before Month 12

	Adolescent Patients				
	A*	B	C	D	E
Treatment received at	Baseline, and Months 1 and 2	Baseline and Month 9	Baseline, and Months 1 and 3	Baseline and Month 1	Baseline, and Months 1, 2, 4, and 5
No. of injections	3	2	3	2	5
Mean	3				
Range	2–5				

* Patient A also received four ranibizumab 0.5 mg injections in the fellow eye at Months 3, 4, 7, and 10 for CNV secondary to Best disease (the same CNV etiology as in the study eye).

### Safety

No serious AEs, severe AEs, or AEs were suspected to be related to the study drug. Ocular and nonocular AEs of the study eye and the fellow eye experienced by the patients are summarized in Table 5. No clinically notable abnormal vital signs or patients with intraocular pressure ≥30 mmHg in the study eye at any time after baseline were reported. No deaths or cases of endophthalmitis were reported.

**Table 5.** Treatment-Emergent Ocular and Nonocular AEs of the Study Eye and the Fellow Eye Over 12 Months by Preferred Term

AEs, Preferred Term	Adolescent Patients*		
	A	B	C
Ocular AEs			
Study eye	Ocular discomfort†	Conjunctival hemorrhage†	Dry eye†
	Conjunctival hemorrhage†	Visual impairment	Conjunctival hemorrhage†
	Eye pain†		Scratch†
	Ocular hyperemia		
	Eye swelling		
	Vision blurred		
	Conjunctival hyperemia		
Fellow eye	Vision blurred	Visual impairment	—
	Lacrimation increased†		
Nonocular AEs	Headache†	Epistaxis	Toxoplasmosis
	Pyrexia	Abdominal pain lower	Nasopharyngitis
	Dizziness		
	Weight increased		
	Tendonitis		
	Vertigo		

* No AEs were reported in adolescent Patients D and E.

† At least one episode was suspected to be related to ocular injection.

## Discussion

Currently, anti-VEGFs are the first-line treatment for CNV lesions in adults with neovascular AMD and myopic CNV.^9^ In the European Union, ranibizumab was approved in November 2016 to also treat CNV due to other causes in adults.^12^ No clear standard of care or treatment paradigm is established for the pediatric population with CNV, although anti-VEGF agents, laser photocoagulation, and verteporfin photodynamic therapy appear to be effective treatment options in cases with severe vision loss.^8,13–20^

Ranibizumab is a well-established and approved therapy for neovascular AMD and CNV in adults,^9,10,21–23^ and therefore is likely to have a similar beneficial effect in a more diverse population with CNV lesions due to other etiologies. Thus, in the MINERVA study, all adolescents diagnosed with various CNV etiologies received an open-label ranibizumab treatment.

The MINERVA study included five adolescent patients with CNV secondary to idiopathic chorioretinopathy, optic disk drusen, and Best disease. Idiopathic CNV occurs in the absence of any known associated condition.^18^ Optic disk drusen usually simulate papilledema, but the associated hemorrhages resulting from the CNV are largely responsible for the central vision loss in children and adolescents.^18^ Best disease is characterized by vitelliform lesions of the central macula and electro-oculographic abnormalities.^19,20^ Choroidal neovascularization secondary to Best disease is often associated with acute vision loss, but some cases may also progress to disciform scar vision loss.^19,20^ In MINERVA, the adolescent patients with these CNV lesions reported a mean visual acuity gain of +16.6 letters at Month 12 with ranibizumab treatment. Few pediatric cases with these CNV lesions have also reported improvement of visual acuity with other treatment options, such as laser photocoagulation and verteporfin photodynamic therapy.^2,3,8,18–20^

Limited studies have reported the off-label use of anti-VEGFs in children with CNV irrespective of the underlying etiology.^13–17,24–26^ Vision improvement has been reported in some patients with CNV aged 9–11 years after the administration of a few (range, 1–3) intravitreal bevacizumab injections.^24–26^ In a 14-year-old girl with CNV due to acute multifocal posterior placoid pigment epitheliopathy, a single intravitreal ranibizumab injection resulted in complete regression of CNV.^13^ Similarly, CNV due to traumatic Bruch membrane rupture resolved after a single ranibizumab injection in a 14-year-old boy.^14^ Choroidal neovascularization due to toxoplasmosis in a 7-year-old patient was successfully treated with ranibizumab and antiparasitic therapy.^15^ Kohly et al^16,17^ described four pediatric patients with CNV, each of whom was treated with different intravitreal anti-VEGF agents: one patient was treated with pegaptanib sodium, two with bevacizumab, and one with ranibizumab.^16^ Visual acuity was improved or maintained after two to five injections in all four pediatric patients.^16^ Short-term results of ranibizumab treatment for CNV due to inflammatory chorioretinal disease and idiopathic CNV in patients aged 10 and 15 years, respectively, have been encouraging in improving visual acuity.^17^

In this subset of the MINERVA study, ranibizumab 0.5 mg treatment over 12 months was beneficial in improving BCVA and stabilizing or reducing CSFT in adolescent patients with CNV. The associated CNV etiologies enrolled in this study were Best disease, optic disk drusen, or idiopathic chorioretinopathy. These are important findings, as the characteristics of CNV may differ between children and adults and may affect the prognosis and natural course of CNV. In few studies, it has been suggested that such differences in characteristics of CNV may lead to more favorable treatment outcomes in younger patients.^1–4^ It should be noted that a mean of only three ranibizumab injections over 12 months was required potentially preventing rapid deterioration of the underlying retinal disease and worsening of vision. These findings from the MINERVA study were consistent with previously published literature in children and adolescents,^13–17^ including a few isolated case reports in which a single intravitreal injection of ranibizumab was reported to be effective in resolving CNV.^27–29^ Moreover, in MINERVA, ranibizumab injection was well tolerated in adolescents, considering the challenges in administration of intravitreal treatment in younger patients. These results reinforce that ranibizumab may be an effective treatment option in adolescents with CNV.^13–17^

In a clinical trial setting, the MINERVA study describes the treatment with ranibizumab 0.5 mg in adolescent patients with CNV of certain etiologies—idiopathic chorioretinopathy, Best disease, and optic disk drusen. The adolescent part of this study entailed several limitations. The sample size was small, and no other CNV etiologies were enrolled. Other limitations included the open-label study design, with no control group and a relatively short follow-up duration of 12 months for the evaluation of retinopathy. However, because CNV is rare in the pediatric population, and owing to ethical considerations in a disease without any standard of care, it was not feasible to conduct a randomized, sham-controlled clinical study. Despite these limitations, it should be noted that all cases were well documented, including the patients' retinal imaging and OCT findings.

Ranibizumab treatment proved to be beneficial for improving visual acuity in these patients with relatively few injections, but in addition prevented the worsening of vision in this pediatric population. This improvement was accompanied by stabilization or reduction in CSFT over the 12-month period. Overall, ranibizumab 0.5 mg was well tolerated, and there were no new safety findings identified up to Month 12. The MINERVA study findings complement the limited available data on the use of ranibizumab for the treatment of CNV with various etiologies in adolescents.
